# Genomics insights into different cellobiose hydrolysis activities in two *Trichoderma hamatum* strains

**DOI:** 10.1186/s12934-017-0680-2

**Published:** 2017-04-19

**Authors:** Peng Cheng, Bo Liu, Yi Su, Yao Hu, Yahui Hong, Xinxin Yi, Lei Chen, Shengying Su, Jeffrey S. C. Chu, Nansheng Chen, Xingyao Xiong

**Affiliations:** 1 Hunan Provincial Key Laboratory of Phytohormones and Growth Development, Hunan Provincial Key Laboratory for Crop Germplasm Innovation and Utilization, Hunan Agricultural University, Changsha, 410128 China; 2 College of Life Science and Technology, Huazhong Agricultural University, Wuhan, 430070 China; 3 National Center for Citrus Improvement, Hunan Agricultural University, Changsha, 410128 China; 4 Wuhan Frasergen Bioinformatics Co. Ltd, 666 Gaoxin Road, East Lake High-tech Zone, Wuahn, 430075 China; 5 Department of Molecular Biology and Biochemistry, Simon Fraser University, Burnaby, BC V5A 5S6 Canada

**Keywords:** *Trichoderma hamatum*, Comparative genomics, Genetic diversity, β-Glucosidase, Cellobiose

## Abstract

**Background:**

Efficient biomass bioconversion is a promising solution to alternative energy resources and environmental issues associated with lignocellulosic wastes. The *Trichoderma* species of cellulolytic fungi have strong cellulose-degrading capability, and their cellulase systems have been extensively studied. Currently, a major limitation of *Trichoderma* strains is their low production of β-glucosidases.

**Results:**

We isolated two *Trichoderma hamatum* strains YYH13 and YYH16 with drastically different cellulose degrading efficiencies. YYH13 has higher cellobiose-hydrolyzing efficiency. To understand mechanisms underlying such differences, we sequenced the genomes of YYH13 and YYH16, which are essentially identical (38.93 and 38.92 Mb, respectively) and are similar to that of the *T. hamatum* strain GD12. Using GeneMark-ES, we annotated 11,316 and 11,755 protein-coding genes in YYH13 and YYH16, respectively. Comparative analysis identified 13 functionally important genes in YYH13 under positive selection. Through examining orthologous relationships, we identified 172,655, and 320 genome-specific genes in YYH13, YYH16, and GD12, respectively. We found 15 protease families that show differences between YYH13 and YYH16. Enzymatic tests showed that exoglucanase, endoglucanase, and β-glucosidase activities were higher in YYH13 than YYH16. Additionally, YYH13 contains 10 families of carbohydrate-active enzymes, including GH1, GH3, GH18, GH35, and GH55 families of chitinases, glucosidases, galactosidases, and glucanases, which are subject to stronger positive selection pressure. Furthermore, we found that the β-glucosidase gene (*YYH1311079*) and pGEX-KG/*YYH1311079* bacterial expression vector may provide valuable insight for designing β-glucosidase with higher cellobiose-hydrolyzing efficiencies.

**Conclusions:**

This study suggests that the YYH13 strain of *T. hamatum* has the potential to serve as a model organism for producing cellulase because of its strong ability to efficiently degrade cellulosic biomass. The genome sequences of YYH13 and YYH16 represents a valuable resource for studying efficient production of biofuels.

**Electronic supplementary material:**

The online version of this article (doi:10.1186/s12934-017-0680-2) contains supplementary material, which is available to authorized users.

## Background

The growing worldwide demand for energy and the desire to reduce dependency on fossil fuels have triggered increased interest in identifying alternative energy resources, especially liquid biofuels, such as bioethanol and biodiesel. Because renewable lignocellulosic biomass is generally considered to be cheaper resource, no competition with agricultural production and cleaner raw material for ethanol production comparing with oil-based fuels [1], efforts in generating liquid biofuels from renewable lignocellulosic biomass have been made.

Biodegradation of lignocellulosic residues is a process that is primarily performed by microorganisms that can enzymatically digest polymeric sugars to capture soluble monosaccharides and disaccharides as carbon sources for energy production. This ability is exploited by biotechnological industries to obtain large quantities of active, stable, and specific enzymes using agricultural waste solids as raw materials [2]. In 2015, the global market for industrial enzymes is expected to reach more than 4 billion dollars [3]. The industrial enzymes market prefers microbial enzymes because they are more stable than enzymes from plants and animals. Fungi are particularly preferred for enzyme production because they are secreted as enzyme complexes that function in a synergistic manner, and their production is a relatively easy and inexpensive [4].

Currently, most kinds of commercial cellulase (including β-glucosidase) are derived from fungi, e.g. *Trichoderma, Aspergillus, Phanerochaete, Schizophyllum and Penicillium* [5]. *Aspergillus niger* is used to produce many pectinases [6, 7] and hemicellulases [8] in industry. *Trichoderma reesei* QM6a was found to be a good producer of cellulose [9]. Due to their efficiency in producing and secreting a broad range of cellulases and hemicellulases, both of these fungi have been the focus of extensive studies on glycoside hydrolase (GH) discovery, and there is a marked effort to understand the regulation of the expression of genes that encoding them.

Species in *Trichoderma* spp. is a widely distributed saprophytic ascomycete and is well known for their biocontrol ability and lignocellulose degradation abilities. Recent genome sequencing projects have targeted eight species [10]: *T. reesei*, *Trichoderma virens*, *Trichoderma atroviride*, *Trichoderma harzianum*, *Trichoderma longibrachiatum*, *Trichoderma asperellum*, *Trichoderma hamatum*, and *Trichoderma citrinoviride*. It was observed that the tropical species *T. reesei* enhances the induction of its entire cellulolytic and hemicellulolytic arsenal when facing temperate *R. solani*, which is a very unlikely prey/host for this species in nature, whereas such a response is not observed for *T. atroviride* or *T. virens*. The presence of a basidiomycete fungus may thus signal the availability of predigested plant biomass to *T. reesei*, consistent with the hypothesis that this species became a saprotroph by following basidiomycetes into their habitat [11].

A striking weakness of the *Trichoderma* system is that many *Trichoderma* strains isolated from the wild lack necessary lignocellulolytic enzymes for efficient bioconversion processes [12], especially β-glucosidases, which are considered key rate- limiting enzymes in the process of cellulose degradation [13]. For example, under cellulase-inducing conditions, the production of secreted β-glucosidase comprises only about 1% of the total *T*. *reesei* cellulase [14], indicating that the hydrolysis of cellobiose constitutes a rate-limiting step during the enzymatic processing of cellulose [15, 16]. Although commercial cellulase is available, many of the most well-known biomass- degrading fungi display low β-glucosidase (cellobiose) activity, thus the initial bioconversion of biomass to sugars remains a key bottleneck in the process of biofuel production. Thus, searching for *Trichoderma* strains with strong β-glucosidase activities is primary importance.

β-Glucosidases (EC 3.2.1.21) are found in all domains of living organisms, where they play essential roles in the removal of nonreducing terminal glucosyl residues from saccharides and glycosides. β-Glucosidases function in glycolipid and exogenous glycoside metabolism in animals, defense, cell wall lignification, cell wall β-glucan turnover, phytohormone activation, and release of aromatic compounds in plants, and biomass conversion in microorganisms. We identified *T. hamatum* strains from cultivated soil in HeJiaqiao, LiLing, Hunan province, China, among which YYH13 exhibited much higher antimicrobial activity against the bacterial wilt pathogen because of its higher expression of specific β-glucanase and chitinases, which play important roles as hydrolytic enzymes during cell wall degradation [17].

In this study, we carried out genome-wide comparative analysis of *T*. *hamatum* and other model organisms with publicly available genomes including *T. atroviride*, *T. harzianum*, *T. reesei*, and *T. viren,* which will help us explain the possible reason for YYH13 and YYH16 genome difference. To examine whether YYH13 has higher cellobiose hydrolyzing efficiency, we subjected YYH13 and YYH16 to exoglucanase, endoglucanase, β-glucosidase activity tests and expression assay of GH1 genes. In total, our results will provide a valuable resource and the genome sequence of *T. hamatum* YYH13 represents a new strain that can be used for further studies on the genetic bases of efficiently degrade cellulosic biomass for biofuel production by the *Trichoderma* species.

## Results and discussion

### YYH13 and YYH16 are two strains of *T*. *hamatum* with different cellulose degradation activity

**Fig. 1:**
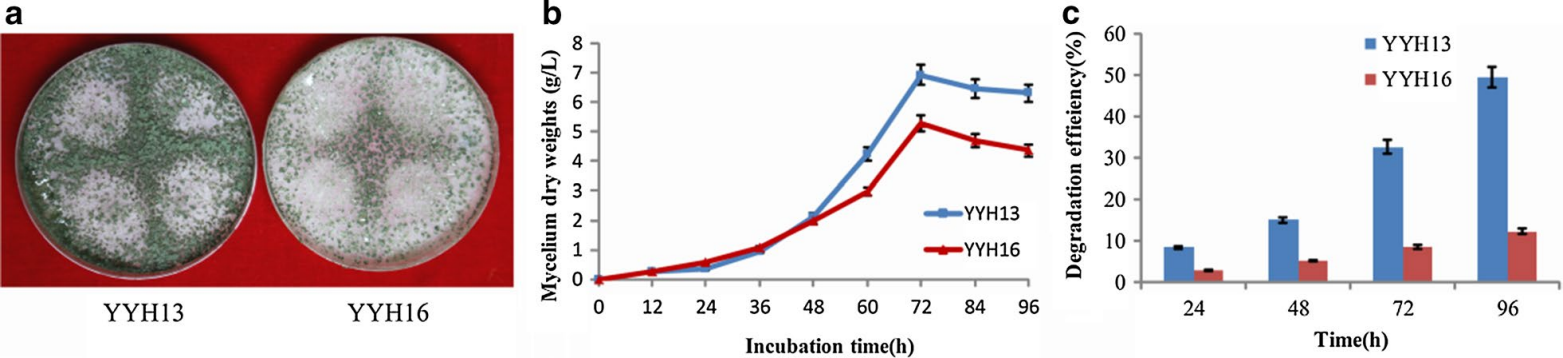
*Trichoderma hamatum* growth natures, and anti-microorganism activity and cellulose degradation induced by YYH13 and YYH16. **a** 72 h cultured *Trichoderma hamatum* in PDA plate. **b** Growth curves of *Trichoderma hamatum*. **c** Filter paper degradation by YYH13 and YYH16 strains. All analysis were performed in triplicate. The *different letters* indicate statistical differences between the different assays (P < 0.05)

*Trichoderma hamatum* have unique mycelium, spore, and colonial morphology [18]. By colonial morphology analysis (Fig. 1a), we found YYH13 and YYH16 strains had moderate colony growth with white and dense colonies. The mycelium was white and the spore heads were green. YYH13 and YYH16 strains isolated in this study showed typical *T*. *hamatum* phenotype. However, these two strains had different mycelial growth when they started at the same spore densities (10^6^ spores/mL), with YYH13 and YYH16 spore density generate difference after 48 h (Fig. 1b).

Many alternative mechanisms can cause microorganism growth inhibition, including mycoparasitism, bacteriolysis, nutrient, and space competition [19]. *Trichoderma* produces many hydrolases that degrade the cell wall, including chitinases, cellulases, xylanase, glucanase and proteases. These enzymes are usually extracellular, of low molecular weight and highly stable. They may be produced in multiple forms or isozymes that differ in size, regulation, and ability. This trait has often been utilized as a means of in vitro screening for biocontrol candidates. Various cell wall degrading enzymes play a very important role in the process of hyperparasitism. Some *Trichoderma* species have strong cellulose-degrading properties because they can secrete an enzyme system capable of degrading crystalline cellulose [20]. For example, *T*. *reesei* QM6a strain possesses a remarkable set of genes encoding hydrolytic enzymes.

We performed cellulose degradation test using filter paper as the substrate. As shown in Fig. 1e, filter paper degradation efficiency of YYH13 at 96 h was 37.14% higher than that of YYH16 at the same time point (*P* < 0.05), YYH13 also showed much stronger capability for the degradation of cellulose (Fig. 1e). The filter paper degradation analysis indicated that the action of its enzymes is very potential in insoluble cellulosic substrates, due to the crystalline structure of filter paper, degradation of the filter paper would imply multiple cellulose activities, including exoglucanase activities because these enzymes work in crystalline regions. In conclusion, we observed that both YYH13 and YYH16 had rapid growth rates with the similar colonal morphologies, and similar growth curves. Despite of these similarities, these two *T. hamatum* strains show significant differences in cellulose degradation activities.

### Genome sequencing and assembly of YYH13 and YYH16

**Fig. 2:**
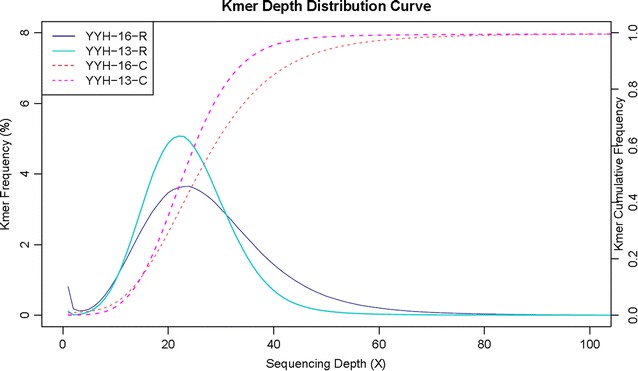
17-mer analysis of YYH13, YYH16, and T. reesei QM6a. A single peak is seen around 60 in YYH13 and YYH16. The longer tail of YYH16 suggesting more repetitive content

To identify the genetic causes of the observed phenotypic differences, we sequenced the genomes of these two *T. hamatum* strains used Illumina Hiseq sequencing platform (Table 1). According to the sizes of assembled genomes of various *Trichoderma* species (Table 2), our sequencing data achieved approximately 292× and 95×, respectively. A 17-mer genomic survey using approximately 70× of data showed that YYH13 had a sharp peak at approximately 60×, indicating low levels of heterozygosity and low levels of repetitive content. Although YYH16 showed a similar low level of heterozygosity, its distribution had a longer tail at higher depths, suggesting more repetitive content (Fig. 2). The K-mer analysis estimated 15% more repetitive sequences than YYH13 (Table 3). We carried out de novo genome assembly to obtain draft genome assemblies using SOAPdenovo. We obtained 608 and 2550 scaffolds with N50 of 578.2 and 41.6 Kb in YYH13 and YYH16, respectively. The final genome assembly sizes for the two genomes were 38.93 and 38.92 Mb (Table 2), respectively, which fell within the known range for the genomes of *Trichoderma* species [21–24].

**Table 1:** Sequencing data size and output quality in YYH13 and YYH16

Type	YYH13 Raw data	YYH13 Clean data	YYH16 Raw data	YYH16 Clean data
Number of reads	62,431,080	58,834,452	20,861,707	19,051,316
Data size	12,486,216,000	11,766,890,400 (94.24%)	4,172,341,400	3,810,263,200 (91.32%)
N of fq1	0.00%	0.00%	0.02%	0.01%
N of fq2	0.01%	0.00%	0.06%	0.00%
Low qual base of fq1: (≤5)	2.73%	0.90%	4.13%	1.61%
Low qual base of fq2: (≤5)	5.07%	1.46%	6.79%	1.88%
Q20 of fq1	95.93%	98.03%	93.11%	96.21%
Q20 of fq2	92.60%	96.63%	90.70%	95.80%
Q30 of fq1	91.76%	94.25%	85.60%	89.43%
Q30 of fq2	87.71%	91.87%	83.15%	88.23%
GC of fq1	45.24%	45.04%	45.14%	44.86%
GC of fq2	45.10%	44.92%	45.04%	44.76%
Error of fq1	0.03%	0.03%	0.05%	0.04%
Error of fq2	0.04%	0.03%	0.06%	0.04%
Discarded reads related to N and low qual	5.76%		8.68%

**Table 2:** Genome assembly and annotation statistics of YYH13, YYH16, and GD12

	YYH13	YYH16	GD12
Scaffolds N50	578,201 bp	41,655 bp	42,825 bp
Scaffolds N90	113,623 bp	9276 bp	10,787 bp
Number of scaffolds	608	2550	1637
Contig N50	79,575 bp	9684 bp	2655 bp
Contig N90	20,512 bp	177 bp	1259 bp
Total size (scaffolds)	38,930,246 bp	38,920,148 bp	36,656,850 bp
Total size (contig)	38,928,694 bp	43,641,621 bp	37,949,230 bp
No. of large scaf (>1 kb)	462	1918	1609
No. of large contig (>1 kb)	1144	7564	9830
G + C content	47.19%	47.93%	48.28%
N rate	0.00%	0.25%	0.62%
Number of CEGs identified	242 (97.58%)	241 (97.18%)	238 (95.97%)
Total protein coding genes	11,302	11,758	11,203
Total gene lengths (exon and intron)	19,416,112	18,964,664	18,415,061
Total exon count	31,674	32,404	33,973
Average exon length	542.43	518.89	457.30
Average exon count per gene	2.80	2.76	3.03
Total introns	20,372	20,646	22,770
Average intron length	109.72	104.16	126.44
Average introns per gene	1.80	1.76	2.03
Average peptide length	506.27	477.38	462.27

**Table 3:** 17-mer analysis using YYH13, YYH16, and reesei sequencing data

Species	Kmer	Kmer count	Kmer depth	Genome size (M)	Revised genome size (M)	Heterozygousity rate (%)	Repeat (%)
YYH13	17	2,603,966,477	60.15	43.29	43.29	0.35	30.13
YYH16	17	2,942,740,366	59.05	49.83	49.82	0.51	46.59
REESEI	17	959,718,356	20.51	46.79	43.45	0.29	30.34

**Fig. 3:**
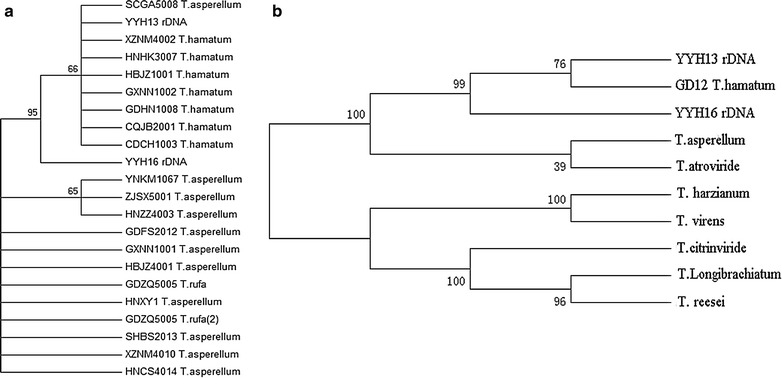
Phylogenetic tree analysis. **a** Phylogenetic tree using ITS1-ITS2 sequences. **b** Phylogenetic tree using* Tef-1*,* Cal-1*, and *Chi18-5*

To confirm that YYH13 and YYH16 are species of *T. hamatum*, we identified the rDNA gene cluster and extracted the sequences for ITS1 and ITS2. Constructing a phylogenetic tree using these ITS sequences with the 92 rDNA *Trichoderma* sequences previously identified [25, 26] showed that YYH13 and YYH16 cluster with known *T. hamatum*, species (Fig. 3a). Similarly, according to three protein coding genes, *Tef*-*1*, *Cal*-*1*, and *Chi18*-*5*, the *T. hamatum*, species cluster together (Fig. 3b). This analysis further confirmed that YYH13 and YYH16 are strains of the *T. hamatum* species.

### Genome annotations of YYH13 and YYH16

To evaluate the completeness of the assembled genomes, we performed a CEGMA (Parra et al. [26]) showed that both YYH13 and YYH16 identified more than 97% of all of the CEGs (complete and partial) (Table 2), higher than that for the published *T. hamatum* GD12, which only identified 95.97%. Thus, our genome assemblies of the two *T. hamatum* strains YYH13 and YYH16 are of good quality. We annotated the genomes for protein coding genes using GeneMark-ES and identified 11,316 and 11,755 genes in YYH13 and YYH16, respectively. The previous *T. hamatum* genome (GD12) was annotated using FgeneSH [21]. To assess the comparability of the two methods, we also annotated the GD12 assembly using GeneMark-ES and compared the annotation results. FgeneSH predicted 10,760 genes, and GeneMark-ES predicted 11,031 genes, for which 10,169 (94% of FgeneSH annotation) genes corresponded to identical annotation structures, and 46 genes were found only by FgeneSH. Thus, we conclude that the FgeneSH gene prediction was comparable to the GeneMark-ES predictions. For consistency, we used the GeneMark-ES annotations for all of the subsequent analyses. The average gene structures (gene size, exon size, and intron size) were also similar among the *T. hamatum* species (Table 2).

We then annotated repetitive elements using RepeatModeler and RepeatMasker and found 1.47% repetitive elements for YYH13 and 1.58% for YYH16, with the most contribution due to simple repeats (Table 4). In terms of transposable elements, *T. hamatum* had similar levels to *T. atroviridae* (0.49%), *T. reesei* (0.57%), and *T. virens* (0.48%) [22]. Finally, the non-coding RNA (ncRNA) annotation tracks were an important contribution to the genome-wide annotation datasets of both YYH13, YYH16, and GD12, not only contributing to the protein-based annotation but also helping to identify annotation errors (Table 5).

**Table 4:** Repetitive element annotation in YYH13, YYH16, and GD12

Type	YYH13	YYH16	GD12
Repeat length	571,404 bp	614,668 bp	478,638 bp
% of genome	1.47%	1.58%	1.31%
DNA transposons	70,804 bp (0.18%)	27,383 bp (0.07%)	54,399 bp (0.15%)
LINE	3710 bp (0.01%)	17,140 bp (0.04%)	7052 bp (0.02%)
SINE	0 bp (0%)	0 bp (0%)	0 bp (0%)
LTR	43,448 bp (0.11%)	44,856 bp (0.12%)	38,856 bp (0.11%)
Satellites	7770 bp (0.02%)	7550 bp (0.02%)	6257 bp (0.02%)
Simple repeats	402,017 bp (1.03%)	432,358 bp (1.11%)	324,187 bp (0.88%)
Low complexity	78,531 bp (0.20%)	83,574 bp (0.21%)	66,153 bp (0.18%)

**Table 5:** Non-coding RNA annotation in YYH13, YYH16, and GD12

RNA type	YYH13	YYH16	GD12
5S_rRNA	56	53	49
5_8S_rRNA	1	0	0
SSU_rRNA_eukarya	1	0	0
snoRNA	20	20	19
Splicesomal RNA	11	14	10
Fungi_SRP	1	1	1
Intron_gpI	1	0	0
RNase_MRP	1	1	1
Total	92	89	80

### Identifying functionally important genes through selection pressure analysis

Selection pressure is an important source for genetic differences that may confer phenotypic differences. By examining the nonsynonymous and synonymous substitution rates of all of the one-to-one ortholog pairs of YYH13, YYH16, and GD12, we found that the majority of the genes (>98%) exhibited Ka/Ks <1, suggesting that most of the orthologs are highly conserved in evolution (Additional file 1). Nevertheless, we found that 131 genes between YYH13 and YYH16, 146 genes between GD12 and YYH16, and 154 genes between GD12 and YYH13 corresponded to Ka/Ks value greater than 1. To screen for YYH13 genes that underwent positive selection, we selected genes that satisfied positive selection criteria between YYH13 and the other two genomes but neutral or purifying selection between YYH16 and GD12. Interestingly, we found 13 genes that satisfied the above condition, one of which (GB7226_YYH13) encodes a putative subtilisin protease, which has been shown to be an exoprotease during cellulose metabolism [23, 24].

Notably, the difference between the percentages of non-synonymous mutations was retained among the three strains, which may be due to the different physiological conditions used for the selection of the strains. The selection could have been stronger for YYH13, resulting in positive selection, and thus preferential retention of non-synonymous SNVs. Moreover, Darwinian selection was tested [27], and the results showed that positive selection drove the evolution of sequences leading to well-known β-glucosidases involved in lignocellulose. Indeed, this study found that YYH13 has 13 genes with Ka/Ks >1, there is an obvious selection pressure will lead to β-glucosidase gene (*YYH1311079*) production diversity and genetic and functional difference.

### Synteny analysis of *T. hamatum* strains

**Fig. 4:**
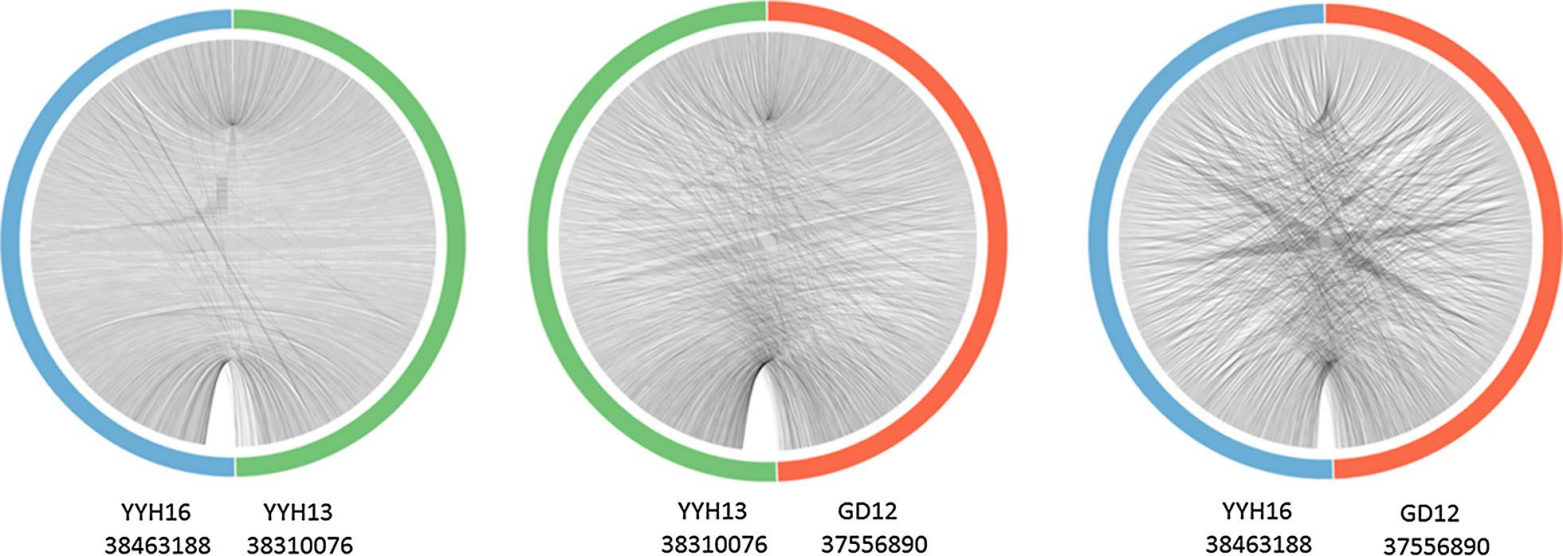
Circos plot of synteny relationships between two genome assemblies. The synteny is based on imperfect synteny blocks identified by Orthocluster. The contigs for the relationships are ordered by the assemblies on the *left*. The number below the strain name is the genome size with contigs that show one-to-one orthologous relationships. Each line in the centre of the *circos plot* represent one synteny block between the two assemblies. A more ordered lines indicates higher level of synteny conservation while a more complex banding pattern represent less synteny conservation

Because YYH13 and YYH16 were isolated from the same location, we expected their genomic structures to be highly similar. We examined synteny relationships using orthocluster based on the gene annotations and one-to-one orthologous relationships identified by Inparanoid, and we found that YYH13 and YYH16 exhibited high synteny levels, whereas YYH16 and GD12 showed the least synteny (Fig. 4). This result is in agreement with the phylogenetic analysis that revealed YYH16 is slightly more distant to YYH13 and GD12, suggesting that YYH16 may have undergone genome rearrangement that caused a decrease in its lignocellulose degradation activity.

As previously argued, high synteny between organisms indicates evolutionary relatedness. Therefore, we expect to find more genes with high synteny than between more distant pairs of species. However, Berlin [28] argued that gene transposition, insertions, deletions, and duplications and rearrangements of chromosome fragments destroy synteny. We found that although some characteristics of the *tri/TRI* cluster have been conserved during evolution of YYH13 and YYH16, the cluster has undergone marked changes, including gene loss or gain, gene rearrangement, and divergence of gene function. In comparison, previous studies [29] have indicated that syntenic gaps in other genomes are enriched in genes that are important for species difference attributes. Although the mechanism and specific biological functions of YYH13 gene duplication have not be clarified, Ambro [30] showed that gene evolution is accelerated to derive new functional genes after gene duplication.

### Strain-specific genes in YYH13, YYH16 and GD12

**Fig. 5:**
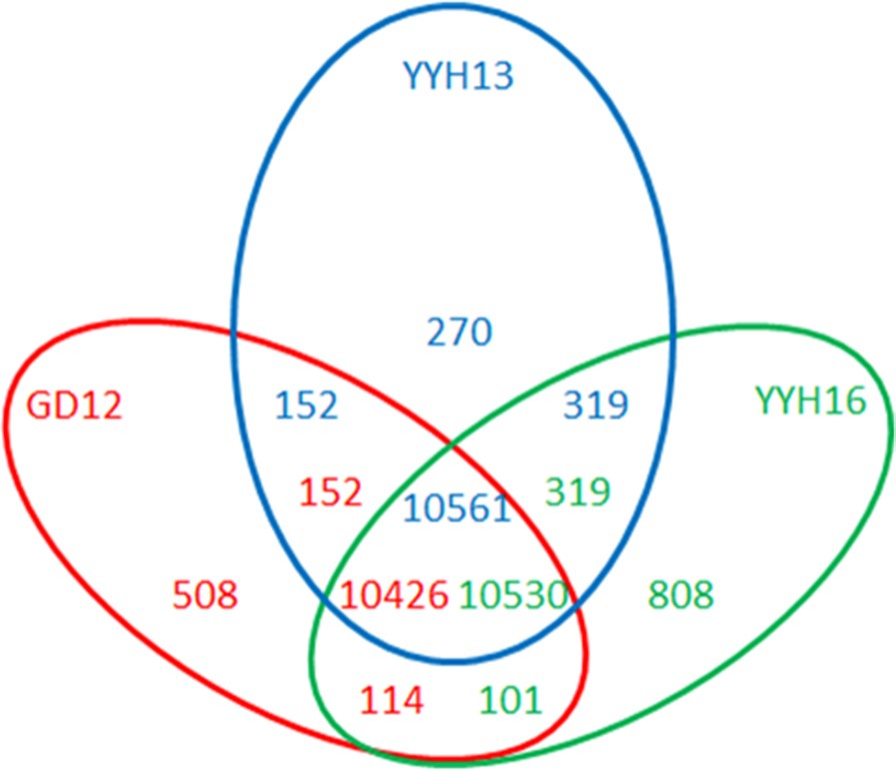
Venn diagram of orthologous relationships identified by Tribe-MCL between YYH13 (*blue*), YYH16 (*green*) and GD12 (*red*). The orthologous relationships includes one-to-one, one-to-many, many-to-one, and many-to-many relationships

One way for obtaining a new phenotype is by acquiring new genes [31]. To examine this possibility, we identified strain-specific genes in YYH13, YYH16, and GD12 by examining orthologous relationships between gene annotations. Although over 90% of the orthologous genes are shared among all three genomes, a small fraction of genes show genome specificity. Based on the GeneMark-ES annotation, we identified 270 genes in YYH13, 808 genes in YYH16, and 508 genes in GD12 that are strain-specific (Fig. 5). We employed a gene revision procedure to ensure that these genes were not identified due to technical errors in gene annotation or inadequate genome assembly. After revision, we found 172 genes in YYH13, 655 in YYH16, and 320 in GD12 that we believe are genome difference with high confidence. However, the majority of these genes do not possess any known functional annotation and only match hypothetical genes in other species (Additional file 2). Of the genes that can be annotated, YYH13 possess a gene with a subtilase domain and another gene with an alpha/beta hydrolase domain, both of which are domains found in many peptidases. In YYH16, we found three genes with helicase domains and two genes with transporter function. The comparison of gene functions in these genome difference genes showed that the functions between the genomes are quite different, suggesting that YYH13 and YYH16 may have undergone strain difference evolution.

In this study, the rate of synonymous substitutions in the YYH13 gene was found to be very small, which generally occur in the process of evolution during a large-scale genome duplication event, indicating that recent duplication has played an important role in the creation of synonymous substitutions. At the same time, the purification selection pressure after YYH13 difference gene duplication was less than gene duplication of communalism, which suggests that the difference of the difference genes is more likely to produce functional variations. Nevertheless, difference genes of YYH16 may be due to functional redundancy, which contribute less to degrading lignocellulose. Similarly, the instability of transposable elements may lead to YYH16 gene rearrangement, and distribution imbalances of insertion sequences may also affect its evolution, leading to difference expression differences among strains.

### Proteases gene family comparison

Proteases are important enzymes that digest and cleave peptides at various levels of metabolism. However, proteases of *Trichoderma* have not been systematically compared at the whole-genome scale. Here, we systematically annotated protease genes in the YYH13, YYH16, and GD12 genomes using the MEROPS database [32]. A large portion (9%) of the secreted protein of *T. harzianum* was identified as proteases when grown on cellulose [33], suggesting its importance in the cellulose degradation process. We annotated the gene and identified 58 protease families in the YYH13 and YYH16 genomes. Of these families, 26 initially showed gene family expansion or contraction. After gene revision, we found 15 gene families that still differ between YYH13 and YYH16 (Table 6). Gene families that have more members in YYH13 include all of the metallopeptidases, whose activity requires metal ions, whereas those in YYH16 are more varied. Genomes show expansions in carboxypeptidases, which are involved in the degradation of barley cell walls by *T. viride* [34].

**Table 6:** Protease family showing difference between YYH13 and YYH16

Family	YYH13	YYH16	GD12	Function	Location of activity
M04	2	1	1	Thermolysin	Endo
M20	3	2	2	Carboxypeptidase	Exo
M24	10	9	9	x-Pro dipeptidase	Exo
M43	4	3	2	Cytophagalysin, pappalysin	Endo
C14	2	3	2	Caspases	Endo
C19	10	11	9	Ubiquitin peptidase	Endo
C26	6	8	7	Gamma-glutamyl hydrolase	Endo
C85	0	1	1	Deubiquitinylating peptidases	Endo
G01	4	5	4	Scytalidoglutamic peptidase	Endo
M28	10	11	10	Aminopeptidases, carboxypeptidases	
M38	6	8	8	Isoaspartyl dipeptidase	Endo
M54	0	1	1	Archaelysin	Exo
S10	5	6	4	Carboxypeptidase Y	
S33	10	14	12	Prolyl aminopeptidase	Exo
S53	11	12	12	Sedolisin	Endo

Location of activity indicates whether the family is an endoprotease or exoprotease

Enzymes: *C* cysteine proteases, *G* glutamic proteases, *M* metalloproteases, *S* serine proteases

However, carboxypeptidases in the *Trichoderma* species with no known identified, thus the roles of the carboxypeptidases of *T. hamatum* in these interactions are still unknown. The proteases of *Trichoderma* spp. and their biocontrol roles have been previously reported [35]. Interestingly, this work describes a protease gene family analysis of *T. hamatum* focusing on biomass degrading activity. Proteases have evolved to utilize different mechanisms for proteolysis [36, 37]. Further studies are needed to understand what causes *T. hamatum* to produce primarily protease-degrading enzymes when grown in the presence of cellulose.

### CAZyme gene family comparison

**Fig. 6:**
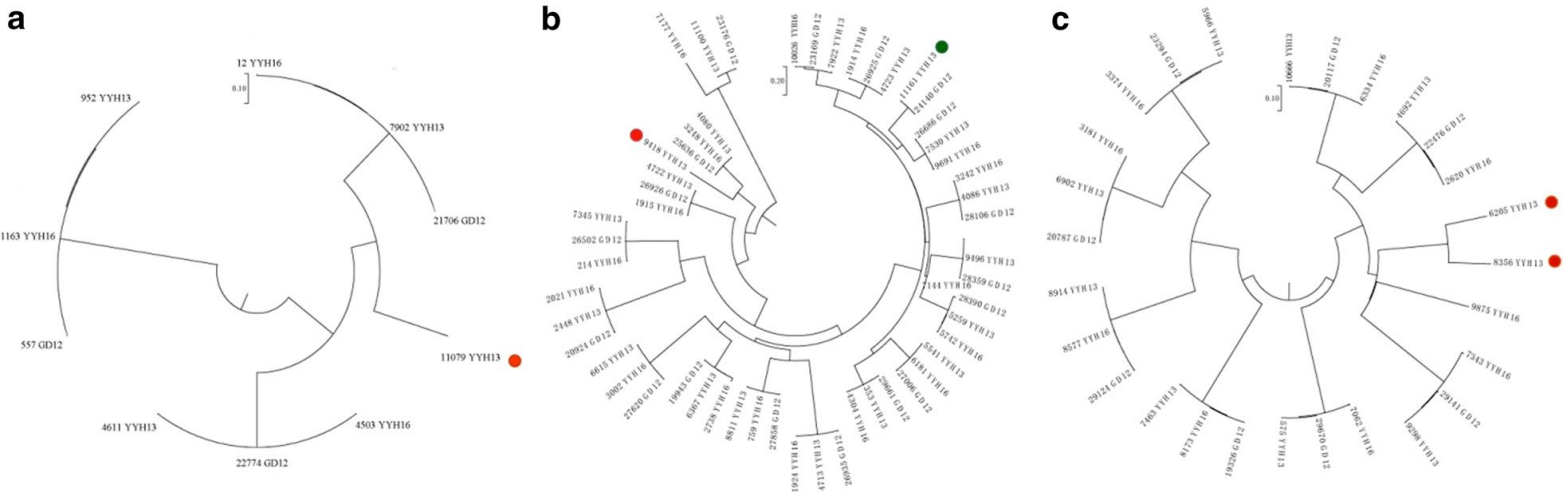
Examples of gene families with gene family expansion in YYH13 or gene family contraction in YYH16. The trees were constructed from **a** GH1, **b** GH3, and **c** GH55 with genes found in YYH13, YYH16, and GD12. The *red dots* indicate gene the expanded genes in YYH13 and the *green dot* indicate sub-family with missing YYH16 likely due to gene family contraction

CAZymes are families of enzymes that degrade, modify, or generate glycosidic bonds [38]. These enzymes, especially those of hydrolytic enzymes, have been associated with the mycoparasitism of *Trichoderma* [39]. Of the 140 CAZyme gene families that we annotated using dbCAN, we initially found 36 families that exhibited gene family expansion or contraction between YYH13 and YYH16. To ensure that the observed differences were not due to technical errors from annotation or genome assembly, we employed the same gene annotation revision to recover any gene annotation that may have been missed. After our gene annotation revision, we found 31 gene families that still showed differences between YYH13 and YYH16, including four CAZyme auxiliary enzyme (AA) families, five carbohydrate-binding modules (CBM) families, three carbohydrate esterase (CE) families, 13 glycoside hydrolase (GH) families, and six glycosyl transferase (GT) families (Additional file 3). In general, YYH13 possesses more GH family members than YYH16. In fact, 10 of the 13 expanded GH family members are in YYH13, including many families of chitinases, glucosidases, galactosidases, and glucanases (Table 7). However, the gene family expanded in YYH16 functions in acetylgalactosaminidase, xylanase and α-glucosidase. All of these 10 GH families lacked clear orthologs in YYH16. The phylogenetic analysis indicated that the additional genes in YYH13 are primarily due to gene family expansion and only three families (GH1, GH3, and GH55) suggest gene family contraction in YYH16 (Fig. 6). The other three families with more members in YYH16 showed YYH16 gene family expansion in GH109 and YYH13 gene family contraction in GH30.

**Table 7:** CAZyme families that show difference between YYH13 and YYH16

Family	YYH13	YYH16	GD12	Function
AA11	4	3	4	Copper-dependent lytic polysaccharide monooxygenases
AA4	2	1	1	Vanillyl-alcohol oxidases
CBM13	7	6	7	Cellulose-binding
CBM18	13	11	9	Chitin-biding
CBM67	3	2	2	l-Rhamnose binding
CE1	21	18	20	Esterases
GT31	5	4	5	Acetylglucosaminyltransferase
GT35	2	1	1	Glycogen or starch phosphorylase
GT69	4	3	3	α-1,3-Mannosyltransferase
GH1	4	3	3	β-Glucosidases, β-galactosidases
GH18	31	30	26	Chitinase, xylanase inhibitor
GH20	4	3	3	Exo-acting β-N-acetylglucosaminidases, β-N-acetylgalactosamindase, β-6-SO3-N-acetylglucosaminidases
GH3	19	17	18	Exo-acting β-d-glucosidases, α-l-arabinofuranosidases, β-d-xylopyranosidases, N-acetyl-β-d-glucosaminidases
GH35	3	2	2	β-Galactosidases
GH43	7	6	6	l-arabinofuranosidases, endo-α-l-arabinanases, β-d-xylosidases, exo α-1,3-galactanase
GH55	10	9	8	Exo-β-1,3-glucanases, endo-β-1,3-glucanases
GH76	9	8	8	α-Mannanases
GH78	5	4	4	l-Rhamnosides
GH88	2	1	2	d-4,5-Unsaturated β-glucuronyl hydrolase
AA1	4	5	2	Ferroxidase
AA5	1	2	1	Galactose oxidase, glyoxal oxidase
CBM21	1	2	0	Granular starch-binding
CBM42	2	3	2	Arabinofuranose binding
CE3	3	4	2	Acetyl xylan esterase
CE5	8	9	8	Acetyl xylan esterase, cutinase
GT2	10	11	10	Cellulose synthase, chitin synthase
GT25	0	1	0	Lipopolysaccharide β-1,4-galactosyltransferase
GT4	5	6	4	Sucrose synthase, α-glucosyltransferase
GH109	17	18	13	α-N-Acetylgalactosaminidase
GH30	5	6	6	Endo-β-1,4-xylanase, β-glucosidase
GH4	0	1	0	α-Glucosidase, α-galactosidase

Function is based on CAZY database annotation

Enzymes: *GH* glycoside hydrolase, *GT* glycosyltransferase, *CBM* carbohydrate-binding module, *AA* auxiliary activity, *CE* carbohydrate esterase

**Fig. 7:**
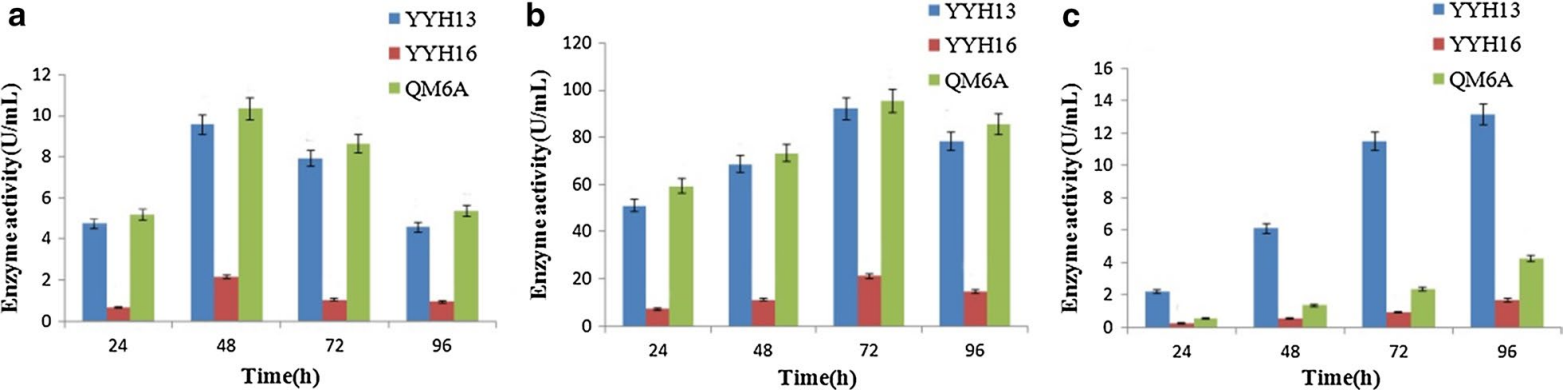
Production of exoglucanase (**a**), endoglucanase (**b**) and β-glucosidase (**c**) of YYH13, YYH16, and *T. reesei* QM6A grown in rice straw. Enzyme activities were determined at 24 h intervals. All assays were performed in triplicate. The *different letters* indicate statistical differences between the different assays (P < 0.05)

Of the GH families, those containing β-glucosidases include GH1, GH3, GH5, GH9, GH30 and GH116. The GH1, GH5, and GH30 β-glucosidases fall in GH Clan A, which consists of proteins with (β/α)_8_ barrel structures. In contrast, the active site of GH3 enzymes comprises two domains, while GH9 enzymes have (α/α)_6_ barrel structures. The mechanism by which GH1 enzymes recognize and hydrolyze substrates with different specificities remains an area of intense study [40]. For the rational design of improved biocatalysts, it is advantageous to work with a well-characterized enzymes or at least enzymes from well-studied families, such as the GH1. These results are consistent with the fact that GH1 enzymes have a large range of potential substrates and specificities, particularly regarding aglycone. To examine whether the activity is indeed higher in YYH13, we subjected YYH13, YYH16, and *T. reesei* QM6a to enzymatic tests on rice straw in exoglucanase, endoglucanase, and β-glucosidase. Our results showed that YYH13 was several-fold higher in activity than YYH16 (Fig. 7).

The saprotrophic species *T. reesei* is a model for studying *Trichoderma* physiology [41]. Comparative genomics showed that YYH13 has a bigger genome than the mycoparasitic species *T. reesei*, suggesting that gene expansion events have occurred in an ancestor of YYH13. YYH16 is a close relative of YYH13, although YYH13 has more lignocellulose degrading related genes, including CAZymes, than YYH16, suggesting that additional saprotrophic gene expansion events occurred in YYH13 after divergence from YYH16. In summary, *T. reesei* is an efficient producer of cellulases and hemicellulases and is used as the major industrial resource of these enzymes [42]. YYH13 is also an efficient cellulase producer. Furthermore, comparing cellulolytic enzymes and hemicellulolytic enzymes indicates that the number of these genes did not reduce but was increased in YYH13. The increase in lignocellulose degrading ability is affiliated with the increase in the number of lignocellulose degrading-related genes. Saprotrophy of plant biomass and the high efficiency of cellulolytic enzymes and hemicellulolytic enzyme production suggest that these enzymes may have been optimized to improve specific activities or expression levels in YYH13. In addition, chitinases, glucosidases, galactosidases, and glucanases are subject to stronger positive selection pressure in YYH13, implying that these enzymes may also play crucial roles in lignocellulose degradation.

The omics data analysis and experimental results showed that YYH13 genome expansion is affected by environmental conditions. To adapt to the specific requirements of the host environment, more genes of YYH13 have been differentiated and have formed multiple gene families. The Red Queen hypothesis [43, 44] considers that microorganisms are constantly faced with a contradiction between evolution and adaptation in the biological environment such that their genomes must be modified and transformed to overcome the contradiction. Phylogenetic analysis revealed that YYH13 mutations function significantly stronger than the effect of homologous recombination and that the classification characteristics and genealogy of YYH13 and YYH16 were shaped by these mutations. Consequently, given the differences in the genomes of strains isolated from the same area and phylogenetic classifications among different geographical regions, notwithstanding the environmental and geographic distribution distance factors, there may be other factors driving the evolution of YYH13, YYH16, and GD12 genomes and their population difference.

### Expression assay of GH1 genes in YYH13 and YYH16

**Fig. 8:**
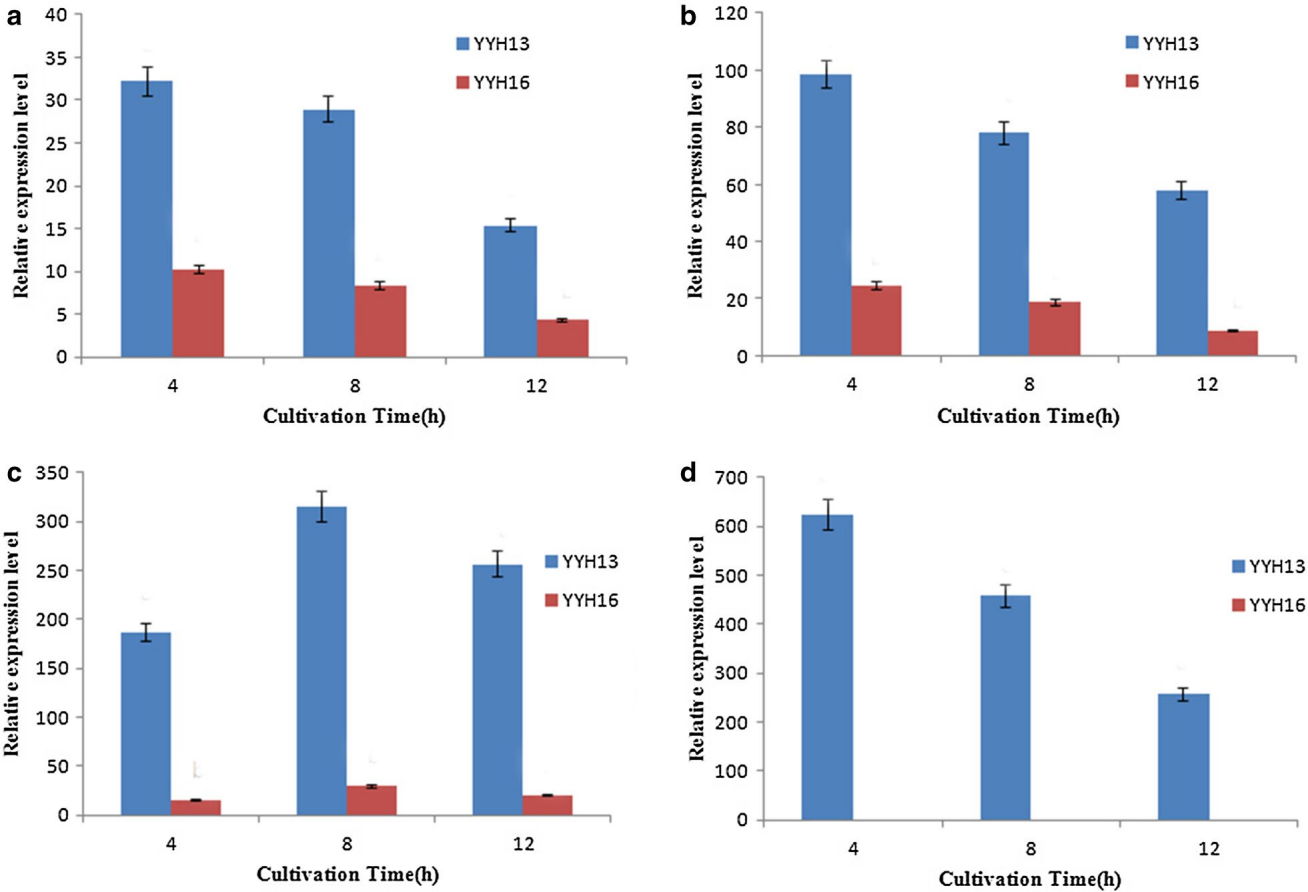
Effects of cellobiose on YYH137902 and YYH1612 (**a**), YYH13952 and YYH1611163 (**b**), YYH134611 and YYH164503 (**c**), YYH1311079 (**d**) beta-glucosidase genes expression in YYH13 (*T. hamatum*) and YYH16 (*T. hamatum*). Genes relative expression levels were determined at different time intervals. All analysis were performed in triplicate. The *different letters* indicate statistical differences between the different assays (P < 0.05)

As shown in Fig. 8, gene expression levels for cellobiose as a carbon source were higher in YYH13 than YYH16. Minimal glucose but no cellobiose was detected in the YYH13 culture, suggesting that cellobiose was readily hydrolyzed to glucose by extracellular β-glucosidases rather than transported into cells. The expression levels of six genes were substantially higher in YYH13 than in YYH16 from 4 to 12 h. The maximum expression levels of *YYH137902* (*YYH1612*), *YYH13952* (*YYH1611163*) and *YYH134611* (*YYH164503*) in YYH13 were three times, four times and 10 times higher than that in YYH16, respectively (Fig. 8). Moreover, the expression of *YYH1311079* was notably higher than other genes when grown on cellobiose in YYH13, indicating stronger degradation levels of cellobiose.

In contrast, we found that the activity of β-glucosidase in YYH13 was significantly higher than QM6A (Fig. 7c). In fact, β-glucosidase is an important component of the cellulase enzyme system that not only participates in cellulose degradation but also plays a key role in hydrolyzing cellulose to fermentable glucose by relieving the inhibition of exoglucanase and endoglucanase from cellobiose. However, it is difficult for *T. reesei* to efficiently convert cellobiose to glucose due to the lack of β-glucosidase, although it is a good producer of cellulase [24].

Cellobiose, which is an intermediate product, is also a strong inhibitor of endoglucanase and exoglucanase and is one of the key bottlenecks in enzymatic hydrolysis [45]. To prevent this inhibition process, the cellobiose unit must be immediately removed. β-glucosidase reduces cellobiose inhibition by hydrolyzing the disaccharide to glucose, allowing cellulolytic enzymes to function more efficiently [46]. Therefore, homologous production and evolutionary studies of the β-glucosidase gene (*YYH1311079*) from the biomass-degrading fungus *T. hamatum* gives new insights into the physicochemical parameters and biodiversity of this family.

### Cloned *YYH1311079* gene and construction of pGEX-KG/*YYH1311079* expression vector

**Fig. 9:**
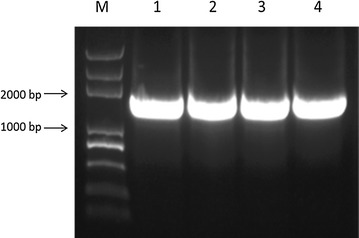
Amplified products of YYH1311079 gene (*M* Trans2K Plus DNA Marker, *1*–*4* PCR products)

**Fig. 10:**
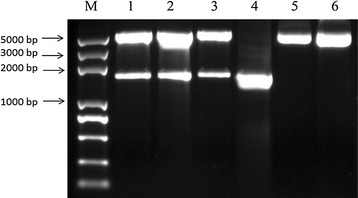
Indentification of pGEX-KG/YYH1311079 recombinant construction (*M* Trans 2K plus DNA Marker, *1*–*3 Bam*HI and *Hind*III enzyme-digested, *4* Target fragment)

In the present study, *YYH1311079* gene fragment of about 1575 bp was cloned according to the YYH13 cDNA library by PCR (Fig. 9). After the double digestion of the recombinant plasmid with *Bam*HI and *Hin*dIII, the result of 1% agarose gel electrophoresis of positive clones showed two specific bands. One was close to the location of plasmid before the digestion, the other had a uniform size as the target gene (Fig. 10). The result of DNA sequencing showed that the inserted fragment was 1575 bp, and when it was matched with the *YYH1311079* gene sequence recorded in the YYH13 genome, the sequencing results were 100% homologous.

*YYH1311079* cDNA clone was inserted in the pGEX-KG, a expression vector at *Bam*HI and *Hin*dIII sites. Following transformation to *Escherichia coli* BL21 (DE3) cells, the recombinant clone was selected and propagated. The recombinant plasmid with *YYH1311079* gene insert was confirmed following the digestion with *Bam*HI and *Hin*dIII which released the fragment of desired 1575 bp. Ampicillin resistance gene and ColE1 origin are provided for selection and maintenance of recombinant in *E. coli*.

The pGEX-KG/*YYH1311079* engineered bacteria was constructed according the antibiotic resistance, colony PCR and sequencing analysis. It indicated that the expression plasmid was constructed correctly. Overall, our results will provide a valuable gene that will be explain whether β-glucosidase is a key rate-limiting enzyme in the process of cellulose degradation. YYH13 strain whether displayed better characteristics in cellulose degradation, and showed great application potentials in ethanol production through degrading renewable lignocellulosic biomass although correlative mechanisms still need further exploration.

## Methods

### Morphological analysis

The isolates were cultured on PDA (Potato Dextrose Agar, Difco) and were incubated in normal light for 3 days at 28 °C. For morphological characterization of *T*. *hamatum*, observations on morphology of mycelium, spore, and colonial were made using Microscopic Imaging System-MVC2000 [17].

### Strains growth conditions and mycelium dry weights determinations

Mature spores of YYH13 and YYH16 strains were collected and re-suspended in sterile distilled water containing 0.05% Tween 20 (Sigma, USA). Spores were counted by haemacytometer. 5 × 10^5^ spores of YYH13 and YYH16 strains were added to 50 mL PDA liquid media respectively, and cultured at 28 °C in the conditions of dark and continuous shaking. For the determination of fungal dry weights, mycelia were collected by two layers of paper filter (Whatman GF-C) after culture of 12, 24, 36, 48, 60, 72, 84 and 96 h respectively. Mycelia were rinsed with distilled water three times and then dried in oven at 60 °C.

### Strains liquid fermentation culture conditions

YYH13 (*T. hamatum*), YYH16 (*T. hamatum*) and QM6A (*T. reesei*) strains at the same growth states were cultured in liquid fermentation medium (NH_4_NO_3_·2 g, KH_2_PO_4_·4 g, MgSO_4_·7H_2_O 0.3 g, CaCl_2_·2H_2_O 0.3 g, MnSO_4_·7H_2_O 0.007 g, FeSO_4_·7H_2_O 0.005 g, NaCl 0.1 g, 1% of rice straw, 1000 mL H_2_O, pH 6.0) at 28 °C and while shaking at 120 rpm for 0, 24, 48, 72, or 96 h. Crude enzyme extract was obtained via centrifugation at 13,000*g*× 10 min at 4 °C, and the supernatants were used for enzyme activity assays.

YYH13 and YYH16 strains at the same growth state were cultured in the minimal medium (NH_4_NO_3_·2 g, KH_2_PO_4_·4 g, MgSO_4_·7H_2_O 0.3 g, CaCl_2_·2H_2_O 0.3 g, MnSO_4_·7H_2_O 0.007 g, FeSO_4_·7H_2_O 0.005 g, NaCl 0.1 g, peptone 3 g, 1000 mL H_2_O, pH 6.0). After 48 h of cultivation at 28 °C while shaking at 120 rpm, the mycelia were harvested and transferred to the same medium containing no peptone, and 1% d-cellobiose was added. The cultures were then incubated at 28 °C while shaking at 120 rpm for 0, 4, 8, or 12 h. All of the assays were performed in triplicate.

### Enzyme assays

All enzyme activities were presented as specific activities using international units (IU) per mL supernatant. The FPase (FPA) activity and endoglucanase (EG) activity were measured by the DNS method with glucose as a standard, as described in [47, 48]. The β-glucosidase activity was determined using p-Nitrophenyl-β-d-glucopyranoside (pNPG) as a substrate based on the reported method by Takashima [49]. The exo-1,4-β-glucanase (CBH) activity was measured as reported by Deshpande [50].

### Sequencing and assembly

The sequenced reads were examined for low quality reads by filtering reads with adaptor sequences of >10% Ns or >50% nucleotides of quality (Q) ≤5. The final output was the clean reads. A genome survey was performed with the clean reads by counting the frequency of 17-mers from 3.8 Gb of data from YYH13 and YYH16. The K-mer frequency was plotted using R.

The assembly was performed using SOAPdenovo [51] with K-mer ranging from 21 to 111, and the assembly with the largest N50 was chosen. Scaffolds that are less than 500 bp were removed in the final assembly.

### Phylogenetic analysis of YYH13 and YYH16

Genomic sequence spanning ITS1 and ITS2 were extracted from a previous sequencing study [21]. A total of 54 ITS1-ITS2 sequences were used as queries in BLAST against YYH13 and YYH16 assemblies. A phylogenetic tree with the ITS1-ITS2 sequences from YYH13 and YYH16 was built by first aligning 92 other sequences from JGI database (http://jgi.doe.gov/) and GenBank (https://www.ncbi.nlm.nih.gov/genbank/). Species recognition in *Trichoderma* is usually based on the application of the genealogical concordance phylogenetic species recognition concept based on the partial genes sequences of translation elongation factor 1ɑ (*Tef*-*1*), calmodulin (*cal1*-*1*), and chitinase 18-5 (*chi18*-*5*) [52]. To further confirmed that YYH13 and YYH16 are strains of the *T. hamatum* species, the concatenated sequence of *Tef*-*1*, *cal1*-*1*, and *chi18*-*5* genes were used to construct a phylogenetic tree as described. Consensus tree was inferred using the neighbour-joining method. Bootstrap analysis was conducted using the MEGA 5.1 (http://www.megasoftware.net/) with 1000 replications to obtain the confidence value for the aligned sequence dataset. A phylogenetic tree was constructed via maximum parsimony.

### Genome annotation

Gene annotation was performed using GeneMark-ES 2.3.e [53] on YYH13, YYH16, and GD12 assemblies. The GD12 assembly and FgeneSH annotation on GD12 was downloaded from JGI. Each gene was annotated for its putative function using GO, the NCBI-nr database, KOG, and KEGG. Putative functional domains were annotated using Pfam (Protein families). Genes with putative CAZyme functions were annotated using dbCAN [54] with version 4 of the database. A valid annotation required database alignment >80 aa, E-value < 1e−5, and percent alignment coverage >30%. Genes with putative protease functions were annotated using the MEROPS database Release 9.13 (Rawlings et al. [32]) with BlastP PID >35%, E-value < 1e−5, and bit score >30. Repetitive element annotation was performed using RepeatModeler and RepeatMasker (www.repeatmasker.org) under the default settings.

### Genome sequence analysis

Orthologous relationships were determined first using Inparanoid [55] under the default settings. Each one-to-one orthologous relationship was examined for possible gene model improvement. Gene model improvement was performed via reciprocal genBlastG [56] comparisons between YYH13 and YYH16. Thus, genBlastG was performed with YYH13 genes as the query and the YYH16 genome as the target, and vice versa. The genBlastG model must lie within the same coordinates as the original gene model. The revised model is from the highest global PID among the three gene pairs (I: Original YYH13 gene and original YYH16 gene; II: Original YYH13 gene and genBlastG model in YYH16 genome; III: Original YYH16 gene and genBlastG model in YYH13 genome).

The gene model revision in GD12 was performed first using the revised YYH13 gene set as the query and further improved using YYH16. If a gene model was improved by both YYH13 and YYH16, only the revision from YYH13 was kept. Finally, the mean PID and standard deviation were calculated based on the revised one-to-one relationships for each pair of genome.

Genome difference genes were identified using Tribe-MCL (inflation value = 1.6) [57] with the original gene set. Each genome difference genes were examined using genBlastG. For each genome difference gene, genBlastG was used against the two other genomes under the default settings. If the genBlastG model and the query showed a global PID ≥ mean PID-2 standard deviations, then the genome difference gene was considered a false positive and filtered.

### Synteny analysis

The synteny blocks between two genomes were analyzed using orthocluster with parameters “-f–rs”. The perfect synteny blocks did not allow for any mismatches. Imperfect synteny blocks were obtained with additional “-i 5–o 5” parameters. The orthologous relationships used as input were the one-to-one relationships based on the Inparanoid results. The Circos diagram was constructed by including only the scaffold containing gene models. The genome on the right was considered the reference, and the genome on the left were reordered.

### Gene family comparison

The gene family annotation for CAZyme and proteases were annotated to the orthologous relationships from Inparanoid. Genomes that were missing orthologous genes in the family were examined using genBlastG revision to ensure the difference observed was not due to misassembly or misannotation. First, if genBlastG was able to produce a gene model in the target genome with percent identity (PID) > mean PID-2 standard deviation, then the model was considered a valid homologous gene. Otherwise, it was considered a low PID and was filtered. If gene family expansion had occurred, a valid genBlastG model may overlap with an existing gene annotation. Thus, if a valid genBlastG model overlapped with an existing gene annotation that already had an ortholog, then the genBlastG model was filtered. The genBlastG models were also annotated using dbCAN and MEROPS, as previously described, and marked as “No annotated function” if the sequence did not pass the annotation criteria.

### Ka/Ks analysis

Synonymous and non-synonymous mutations were determined from pair-wise alignments of revised one-to-one relationships. Ka/Ks ratio was calculated using Ka/Ks Calculator 2.0 using the MYN algorithm.

### Real-time polymerase chain reaction

Mycelia were harvested, frozen and ground in liquid nitrogen. Total RNAs from the mycelia were extracted using TRIzol (Invitrogen, USA), and polyA mRNAs were purified using a PolyATract mRNA Isolation System (Promega, Madison, WI) according to the manufacturer’s instructions. All cDNAs were synthesized via reverse transcription reaction performed using ReverTra Ace (Toyobo, Japan) at 42 °C for 1 h and then 85 °C for 15 min to stop the reaction. The standard protocol was 95 °C for 10 min followed by 40 cycles at 95 °C for 10 s and 59 °C for 50 s. All reactions were performed in triplicate. The *GAPDH* was used as internal reference gene. GH1 family beta-glucosidase genes (*YYH137902*, *YYH13952*, *YYH134611*, *YYH1311079*, *YYH1612*, *YYH1611163*, *YYH164503*) were classified using dbcan analysis system, including *YYH1311079* was specific gene, *YYH137902* and *YYH1612*, *YYH134611* and *YYH164503*, *YYH13952* and *YYH1611163* were between homologous genes. qRT-PCR was performed using PikoReal 96-well thermal cyclers (Thermo, USA) with primers and temperatures as described in Additional file 4.

### Cloning and construction of recombinant plasmid expression vector

*Escherichia coli* strains BL21 (DE3) (Invitrogen, Carlsbad, CA, USA) were used for cloning and expression experiments. *E. coli* strains were grown in Luria–Bertani (LB) broth or on agar plates at 37 °C. Ampicillin (Sangon Biotech, Shanghai, China) was used in growth media when required. The vectors pGEX-KG (Takara, China) was used for polymerase (Additional file 5) chain reaction (PCR) cloning. The coding sequence of YYH1311079 was amplified by PCR using a sense primer (5′-CGCGGATCCATGTCCAAAGAGGCGTC GATGTTC-3′) and an antisense primer (5′-CCCAAGCTTCTATATCCCTCTGCGC CTGGCAAAAG-3′) with *Bam*HI and *Hind*III restriction enzyme sites (underlined), respectively. The protocol is an initial denaturation at 95 °C for 1 min followed by 30 cycles of amplification (95 °C for 10 s, 58 °C for 50 s, and 72 °C for 2 min) and an additional extension step at 72 °C for 10 min. Two white single colonies were selected and inoculated to 5 mL LB culture solution containing 5 µL 100 μg/mL ampicillin and underwent shake culture at 37 °C overnight. The plasmid DNA was extracted using alkaline lysis, and underwent two single digestions with *Bam*HI and *Hin*dIII respectively, and then electrophoresis with 1% agarose gel was done to identify the positive clone. Some of the constructed pGEX-KG/YYH1311079 expression plasmid were sent to Shen Zhen HuiDa an corp in China for sequencing.

## Additional files

**Additional file 1.** Ka/Ks calculations for each orthologs pair between YYH13, YYH16, and GD12.

**Additional file 2.** Genome difference genes annotated with NCBI NR, GO, KEGG, KOG, and Pfam.

**Additional file 3.** CAZyme families that; show difference in member numbers between YYH13 and YYH16.

**Additional file 4.** Primers used in qRT-PCR for validation of differentially expressed genes.

**Additional file 5.** Primers used in PCR for amplified products of *YYH1311079* gene.

## Abbreviations

pNPGase: the β-glucosidase activity
pNPCase: the CBH activity
CMCase: the CMC activity
FPase: the filter paper activity
PDA: potato dextrose agar
IU: international units
pNPG: p-nitrophenyl-β-d-glucopyranoside
PCS: the pretreated corn stover
GAPDH: glyceraldehyde-3-phosphate dehydrogenase
